# Erich Meyerhoff: a man for all medical librarians

**DOI:** 10.5195/jmla.2020.873

**Published:** 2020-01-01

**Authors:** Judith Messerle, Lucretia W. McClure

**Affiliations:** Director (Retired), Countway Library of Medicine, Harvard Medical School, Boston, MA, jmesserle@frontiernet.net; Medical Librarian Emerita, Edward G. Miner Library, University of Rochester Medical Center, Rochester, NY, and Special Assistant to the Director, Countway Library of Medicine, Harvard Medical School, Boston, MA

## Abstract

Erich Meyerhoff—recipient of the Marcia C. Noyes Award, Janet Doe Lecturer, and Fellow—was one of the Medical Library Association’s (MLA’s) most illustrious members who contributed to the welfare of MLA and its members throughout his long life. The authors review his life and significant contributions to the health sciences library profession. Erich was a friend and mentor to countless medical librarians and was truly a man for all medical librarians.

For the first time in the history of the Medical Library Association (MLA), the Fellows of the association authorized a festschrift to honor one of its own, Erich Meyerhoff (1919–2015). It is an honor not given lightly. Rather, it speaks to the many ways in which Erich influenced the association and in particular contributed as a mentor to and example for the well-being and success of members who were his colleagues, new and old. He was a friend to every member.

Who is this man and why do we celebrate him? Erich was born in Braunschweig, Lower Saxony, Germany, in 1919 and was sent to New York in l935 by his parents at the young age of sixteen. After a tour of duty in the US Army, he went to the City College of New York, where he received a bachelor’s of science (BS) in science. That was followed by a master’s degree in social work from the New York School of Social Work (now Columbia University School of Social Work) in 1949. Immediately thereafter, he received a master’s in library science from Columbia University in 1950. He had found his niche in life in a field where he would push the margins and join a revolution in how libraries, particularly medical libraries, would operate into the future [1].

His earliest library assignment as reference librarian at the College of Physicians and Surgeons at Columbia University in New York started a long-lived commitment to medical libraries. Six years after his appointment at Columbia, he became librarian and assistant professor, Health Sciences Library, Down State Medical Center of the State University of New York (SUNY) (1957–1961) [2].

Things were changing in the medical library field and in library management in general. As Erich himself describes in his oral history:

It was a time of great expectations and of active change. It was a time of subjecting library practices to intense scrutiny and the application of rational examinations of library practices, goals and missions. There were people like Fred Kilgour, the librarian at Yale Medical College, already at work programming the foundations of what became the Online Computer Library Center (OCLC). Thomas Fleming of Columbia University was conducting substantial new studies of journal use. Brad Rogers at the National Library of Medicine had committed considerable resources and taken considerable risks in developing MEDLARS, the first computer-aided indexing of the journal literature of medicine and its computer-aided periodical retrieval. [3]

In the midst of that exciting era, medical libraries in New York were experiencing chronic problems with storing the prolific literature being created in medicine and biomedical sciences. Erich joined ongoing discussions about the creation of a new library center that would accommodate long runs of journals and make the materials available to participating libraries as a central repository and delivery service. This central repository concept became a reality in 1960 with the purchase of a building in New York City. Erich was to become the founding director in 1961, responsible for developing all the operating philosophies, services, policies, and procedures for the new Medical Library Center of New York (MLCNY). It was a revolutionary concept for New York medical libraries and changed the practice of all the participating libraries, including many hospital libraries. It was new ground, developed and delivered by Erich while dealing with the politics of collaboration in the midst of competition and providing service at a price that made cooperation affordable [4].

After leaving MLCNY, Erich went on to become librarian of the Health Sciences Library, SUNY at Buffalo (1967–1970). There, he participated as a founding member of the nascent SUNY Biomedical Communications Network (1968–1977), one of the earliest attempts to facilitate interlibrary loan communication and information access via computer [5]. He was drawn back to his city of New York to take a position as librarian, Cornell University Medical College (1970–1977), and was promoted to assistant dean for information services in 1977, a position he held until 1986.

Retirement from Cornell did not slow Erich down. He became the chief of library service at the Veteran’s Administration at New York Medical Center, where he served until 1988 before becoming assistant adjunct curator, Ehrman Medical Library Archives at the New York University School of Medicine, where he worked until “real” retirement.

His work on the MLCNY, in many ways defines why we celebrate Erich. He was a man for the common good. With a twinkle in his eye, he could be most persuasive and accommodating and always brought out the best in everyone. Erich was a strong proponent of the right thing but was able to present that point of view in a manner that was not inflammatory or controversial. He was not afraid to speak up when necessary but also knew when to keep his peace. This was evident at MLA business meetings, where he could be counted on to keep an MLA Board accountable, having read committee reports and proposals of the association. Erich was a doer. He got things done, and those things mattered.

We recognize Erich because he was a friend to all. He enjoyed mentoring younger members of the association and members of his various staffs, encouraging them on their paths, listening to their stories, and making them feel they could make a difference. He was kind and generous with his time and his energies, always working for the common good. There are many today who can recount their “Erich stories” of what he did for them.

We honor Erich because he himself was an inspiration. His Janet Doe Lecture took on the formidable task of analyzing the foundations of medical librarianship. It was a scholarly review that reflected his attention to the larger world of librarianship and its daily practice [6]. Earlier, writing about MLA’s performance in the realm of continuing education, he made forthright recommendations for change, while saluting efforts of the past [7].

Erich was not a bystander in life. He showed up. He was active in MLA, serving on the Board of Directors (1973–1976) and serving as chair of the Ad Hoc Committee to Review the Goals and Structure of MLA (1968–1972). He was active in the New York-New Jersey Chapter of MLA and the Upstate New York and Ontario Chapter, arriving by bus to join the activities. His interest in medical history included MLA’s History of the Health Sciences Section and the American Association for the History of Medicine, where meetings of these groups afforded him many opportunities for making friends and expanding his knowledgebase. For Erich, these meetings were an important part of his life, and he continued to attend them until well into his nineties.

During his lifetime, Erich was awarded MLA’s highest honors: the Janet Doe Lectureship (1977), Fellowship (1985), and the Marcia C. Noyes Award (1997). In 2016, the association renamed the MLA award that “recognizes and stimulates interest in the history of the health sciences” as the Erich Meyerhoff Prize to be given annually for the best unpublished paper in the history of medicine. Erich was also recognized as a Fellow of the New York Academy of Medicine and a Fellow of the American Association for the Advancement of Science

With Erich’s passing in 2015, MLA lost a remarkable intellect and a warm, caring, and witty friend. For these things, we honor him.

**Judith Messerle, AHIP, FMLA** (corresponding author), jmesserle@frontiernet.net, Director (Retired), Countway Library of Medicine, Harvard Medical School, Boston, MA

**Lucretia W. McClure, AHIP, FMLA,** Medical Librarian Emerita, Edward G. Miner Library, University of Rochester Medical Center, Rochester, NY, and Special Assistant to the Director, Countway Library of Medicine, Harvard Medical School, Boston, MA

## References

[1] Greenberg SJ (2016). Erich Meyerhoff, AHIP, FMLA, 1919–2015: a remembrance [obituary]. J Med Libr Assoc.

[2] Medical Library Association MLA Fellow: Erich Meyerhoff [Internet].

[3] Medical Library Association, Oral History Committee Interview about the Medical Library Center of New York with Erich Meyerhoff. Interview conducted by Rachael K. Anderson on 7 Aug 2003.

[4] Meyerhoff E (1963). The Medical Library Center of New York: an experiment in cooperative acquisition and storage of medical library materials. Bull Med Libr Assoc.

[5] Bridegam WE, Meyerhoff E (1970). Library participation in a biomedical communication and information network. Bull Med Libr Assoc.

[6] Meyerhoff E (1977). Foundations of medical librarianship. Bull Med Libr Assoc.

[7] Meyerhoff E (1963). Continuing education of medical librarians: evaluation of the association’s past performance and suggestions for the future. Bull Med Libr Assoc.

